# Extracellular Vesicles in the Biology and Liquid Biopsy Diagnostics of Pediatric High‐Grade Glioma – Emerging Findings and Opportunities

**DOI:** 10.1002/jex2.70152

**Published:** 2026-06-01

**Authors:** Samia Jahouri, Laura Montermini, Nadim Tawil, Mahsa Jalali, Livia Garzia, Nada Jabado, Janusz Rak

**Affiliations:** ^1^ Department of Pediatrics McGill University Montreal Quebec Canada; ^2^ The Institute/Research Institute of the McGill University Health Centre Montreal Quebec Canada; ^3^ Division of Clinical and Translational Research McGill University Montreal Quebec Canada

**Keywords:** extracellular vesicles and particles, liquid biopsy, oncohistones, oncogenes, pediatric high‐grade glioma

## Abstract

Liquid biopsy has emerged as an important diagnostic paradigm impacting care across multiple cancer indications. This is also an area of great interest in pediatric solid tumours, including those affecting the central nervous system (CNS). Of special concern are pediatric high‐grade gliomas (pHGGs) due to the associated diagnostic complexities, poor outcomes and difficult access to tumour tissue, especially for midline lesions. In this commentary we discuss the applicability of different liquid biopsy analytes accessible in cerebrospinal fluid or blood to detect, monitor or molecularly diagnose pHGG, concentrating on the emerging biology of extracellular vesicles and particles (EVPs), as both biological mediators and sources of diagnostic information. We highlight the role of oncogenic pathways in shaping the profiles of EVPs in brain tumours, and we point to diagnostic opportunities associated with the discovery of oncohistones, as possible modulators of EVP landscapes in pHGG.

## 1 Introduction—The Daunting Reality of Pediatric High‐Grade Glioma

Primary tumours of the central nervous system (CNS) occur in children at relatively low incidence, but they are the most important cause of cancer‐related mortality in the pediatric population, surpassing those of leukemia (Girardi et al. 2019; Thorbinson and Kilday 2021). The cited estimates of pediatric CNS tumour incidence range from approximately 6 cases per 100,000 US residents (Price et al. 2025) to 36 cases per million of North Americans, with a five‐year survival rate of less than 20% (Jones et al. 2012). This translates into over 23,000 deaths per year worldwide (Lu et al. 2023).

These sobering numbers reflect the considerable challenges associated with managing neoplastic lesions within the CNS in children, which is further compounded by the increasing diversity among these disease entities (Capper et al. 2018), as reflected by the recent WHO 2021 classification (d'Amati et al. 2024). Amidst this complexity, the pediatric diffuse high‐grade gliomas (pHGGs) account for approximately 10% of all cases, albeit these are cancers associated with particularly dire outcomes (Jones et al. 2017; Damodharan and Puccetti 2023). Indeed, pHGG remain largely incurable despite the wealth of knowledge as to their molecular underpinnings (Capper et al. 2018; Schwartzentruber et al. 2012; Sturm et al. 2023), cellular origin (Jessa et al. 2022; Chen et al. 2020; Furst et al. 2024), immune microenvironment (LaBelle et al. 2025; Ross et al. 2021), vascular peculiarities (Wei et al. 2021), potential therapeutic vulnerabilities (Damodharan and Puccetti 2023; Yoel et al. 2024; Monje et al. 2025) and other key biological properties. While undoubtedly a significant progress is being made, new paradigms (Jones et al. 2017) and sustained efforts are clearly needed to radically change these dismal outcomes.

One research direction that may hold promise in this regard is to better understand, diagnose and therapeutically exploit the heterogeneity of pHGGs including their dynamic cellular ecosystems (Chen et al. 2020). While the evolution, progression and invasion of pHGGs implies the existence of a network of cell‐cell interactions little is known about the underlying mechanisms and the related roles of mobile cellular fragments known as extracellular vesicles and particles (EVPs) (Rak 2013). EVPs are also of diagnostic interest as their release by cancer, stromal and immune cell populations results in their entry into, and accessibility within, biofluid spaces, such as cerebrospinal fluid (CSF), or blood. Tapping into this flux of unique biological material reflecting the cellular composition and molecular make up of the disease is among the focal points in liquid biopsy research and an area of potential applications in pHGG diagnostics (Al‐Nedawi et al. 2008; Skog et al. 2008; Rak and Strzadala 2023). In this commentary, we will survey the rationale and emerging, albeit still preliminary, data, as to the potential role of EVPs in pHGG progression and in the context of prospective liquid biopsy applications.

## 2 The Emerging Molecular Landscape of Pediatric High‐Grade Glioma

In view of their aggressive clinical course, infiltrative growth pattern and cellular characteristics pHGGs are classified as grade IV brain tumours reminiscent of adult glioblastoma (GBM) and high‐grade glioma (HGG) (Wen et al. 2020). This apparent relatedness implied in the earlier literature has recently been radically revised due to developments that positioned pHGGs as distinct disease entities fundamentally different from their adult counterparts, a change illustrated by successive revisions of their WHO classification in 2016 (Louis et al. 2016) and 2021 (d'Amati et al. 2024). Seminal in this regard were studies on the genetic underpinnings of pHGG (Schwartzentruber et al. 2012), which brought to light the new cellular transformation paradigm. Thus, it became clear that in a large proportion of pHGGs the onset of malignancy involves oncogenic mutations of chromatin organizing histone genes, especially histone H3, leading to large‐scale reprogramming of the cellular epigenome (Furst et al. 2024). Among such transforming ‘oncohistone’ mutations the most studied include *H3.3K27M, H3.3G34R/V*, and *H3.1K27M*. The expression of these mutant histones in oligodendrocyte or interneuron precursor cells in the developing brain (Jessa et al. 2022; Chen et al. 2020) results in a stalled differentiation program due to impairments in the distribution of H3K27me3 repressive marks across cellular epigenome (Andrade et al. 2023). The resulting sustained stemness‐like state, whether induced by oncohistones (Andrade et al. 2023), or by other alterations (Korshunov et al. 2017), acts in concert with additional mutations to drive an aggressive cellular phenotype, tumorigenicity and pHGG progression (Furst et al. 2024; Wu et al. 2012).

These molecular findings enabled identification of four different subtypes of pHGG, including: (i) Diffuse midline glioma (DMG) with *H3K27M* alteration; (ii) Diffuse hemispheric glioma (DHG) with *H3G34R/V* mutation; (iii) Diffuse pediatric‐type high grade glioma with *H3K27*‐wild type and *IDH*‐wild type genes and (iv) Infant‐type hemispheric glioma (d'Amati et al. 2024). Each of these entities is associated with a unique repertoire of additional molecular alterations, distinct biological and clinical characteristics, as well as not fully understood range of therapeutic vulnerabilities (Damodharan and Puccetti 2023).

The most dismal in this regard are DMGs harbouring *H3K27M* mutation, and previously often referred to as diffuse intrinsic pontine glioma (DIPG). These tumours occur in children at the median age of 6–7 years and are inoperable because of their location in vital midline brain structures (pons, thalamus cerebellum, spinal cord). DMGs lead to patient demise within 9 to 15 months, depending on the existence of either *H3.3K27M* (*H3F3A*) or *H3.1K27M* (*HIST1H3BK27M*) mutations, respectively (Furst et al. 2024). Oligodendrocyte precursor (OPC)‐like cells were originally suggested as cells of origin for DMG (Monje et al. 2011) and later found to sustain *H3K27M* mutations (Filbin et al. 2018). The oncogenic effects of these mutations involve functional disruption in the activity of the polycomb repressive complex 2 (PRC2) and its catalytic subunit, enhancer of zeste homolog 2 (EZH2), acting as histone demethylase modifying chromatin configuration. This effect leads to the aforementioned impairment of cell differentiation pathways and enforcement of cell stemness (Andrade et al. 2023). Similar mechanisms may also be triggered by the expression of EZH Inhibitory Protein (EZHIP/CXorf67/CATACOMB) that acts as PRC2 inhibitor in a subset of DMG tumours, posterior fossa ependymomas (PFAs) and in other rare cancers (Pun et al. 2023; Cassim et al. 2024). The role of PRC2 inhibition in cellular transformation by oncohistones has been further documented by pivotal mechanistic studies (Lewis et al. 2013).

The impact of enforced cellular stemness and multipotentiality in brain tumours may not be limited to DMG, as glioma stem cells have been extensively characterized in HGG (Singh et al. 2004; Mao et al. 2013; Bao et al. 2006) and in some cases linked to the vascular microenvironment (Calabrese et al. 2007). Moreover, glioma cell populations masquerading as either endothelial cells (Ricci‐Vitiani et al. 2010; Wang et al. 2010) or pericytes (Cheng et al. 2013) have been reported and implicated in neo‐angiogenesis and other vascular anomalies reported in HGGs (Jain et al. 2007), albeit not without some controversy (Rodriguez et al. 2012; Carlson et al. 2021). Recently, single cell profiling studies led to a model of phenotypic heterogeneity, plasticity and spatial organization among diverse cellular states within the complex multicellular ecosystem of HGG (Neftel et al. 2019; Nomura et al. 2025), and a unique architecture of pHGG cell populations was found to be associated with *H3K27M* mutations (Tirosh and Suva 2024; Suvà and Tirosh 2020). The *H3.3K27M* mutant DMGs often carry additional mutations in *TP53*, *FGFR1A*, *PDGFRA*, *EGFR*, and *CCND2* genes, while *H3.1K27M* lesions may be associated with mutant *PI3K*, *PPM1D*, and *ACVR1* (Furst et al. 2024). Subclonal mutations in *ATRX* were also found in a subset of *H3.3K27M* mutant thalamic tumours (Pathania et al. 2017) and in some tumours occurring in the pons (Haase et al. 2018).

In contrast to DMG, DHG lesions driven by the *H3.3G34R/V* mutation are restricted to brain hemispheres and affect mostly adolescents and young adults. As all oncohistone‐driven tumours, DHGs are deadly with predicted median survival of only 22 months. In addition to oncohistones, these tumours may also carry *TP53* and *ATRX* mutations along with methylated *MGMT* promoter (Furst et al. 2024). In this setting malignant process is often exacerbated by alterations in the three‐dimensional chromatin structure resulting in overexpression, mutation and oncogenic activation of PGDFRA (Chen et al. 2020), while the absence of TP53 and growth factor receptor mutations signals a somewhat less aggressive course of the disease (Pathania et al. 2017). DHGs are thought to originate from GABAergic interneuron progenitors (Chen et al. 2020) and exhibit distinctive features of the tumour microenvironment (TME) as exemplified by a unique neovascularization pattern (Wei et al. 2021) reminiscent of vasectasia found in adult mesenchymal GBM models (Spinelli et al. 2024).

While oncohistones drive progression of over 80% of pHGGs, tumours in this category may also arise due to other mutations. Thus, diffuse pediatric‐type HGG, H3‐wild type and IDH‐wild type occur typically in younger children (3–5 years old) affecting hemispheric or midline structures with lesions often expressing oncogenic EGFR, PDGFRA, or MYCN (Korshunov et al. 2017). Oncohistone mutations are also absent in infant‐type diffuse hemispheric gliomas, which instead harbour fusions of receptor tyrosine kinase (RTK) genes (Furst et al. 2024). All pHGGs are characterized by unique methylation signatures that help distinguish these tumour entities from other lesions and among their subtypes (Louis et al. 2021) (Capper et al. 2018; Tran et al. 2024)

## 3 Cellular Complexity and Intercellular Interactions in High Grade Brain Tumours

In addition to inter‐individual diversity of pHGG, each tumour also forms a complex cellular ecosystem that creates conditions for intimate cell‐cell contacts and interdependencies, potentially impacting the disease pathogenesis. Thus, implicitly, interactions between cancer cells and their surroundings occur during pHGG invasion and formation of infiltrative growth patterns (Tari et al. 2022). Glioma cells also interact with neurons (Zhang et al. 2024) recruit different vascular cell populations (Wei et al. 2021) mobilize myeloid cells and drive formation of a complex TME (Liu et al. 2022), in which interactions may also occur between different subsets of cancer cells (Liu et al. 2022; Chanoch‐Myers et al. 2026).

While the full spectrum of this communication web is still to be fully unveiled, studies on adult‐type HGGs offer a useful reference point. Thus, as mentioned, HGGs are often described as mosaics of heterogeneous cancer cell subsets (Patel et al. 2014), maintained in dynamic equilibria through a flux of transitory cellular states (Neftel et al. 2019). In this setting, the dominant features reflective of the apparent molecular tumour subtype are often reflective of phenotypic biases imposed on the cancer cell population by specific driver mutations in concert with intrinsic differentiation programs (Neftel et al. 2019) and intricate spatial organization (Greenwald et al. 2024). These complex cellular ecosystems are thought to contain multiple subpopulations of tumour initiating glioma stem cells (GSCs) and their progeny (Singh et al. 2004; Bastola et al. 2020), both involved in reciprocal cell‐cell communication (Wang et al. 2018). These interactive networks orchestrate the collective behaviour of cancer cell populations (Ricklefs et al. 2016), which also engage neurons (Winkler et al. 2023; Venkatesh et al. 2019), immune cells (Wang et al. 2017) and the vasculature (Calabrese et al. 2007; Chanoch‐Myers et al. 2026; Adnani et al. 2022).

The cell‐to‐cell connections within these cancer cell ‘societies’ (Heppner 1989) are achieved through several mechanisms (Broekman et al. 2018). They range from secretion of soluble paracrine factors (Wang et al. 2018), direct cell‐cell contacts, synapse‐like structures, junctions (Broekman et al. 2018), as well as different specialized membrane formations, including long physical bridges known as tumour microtubes (TMs) (Osswald et al. 2015; Hausmann et al. 2023). In addition, both short and long range (even systemic) cell‐cell communication is maintained by different subsets of EVPs (Rak 2013; Spinelli et al. 2024; Broekman et al. 2018) (Mangena et al. 2025). The latter are of special interest due to their increasingly understood biological roles, as well as their ability to enter common fluid spaces, including blood circulation, whereby they may function as systemically acting biological mediators also accessible as liquid biopsy analytes (Ricklefs et al. 2024).

## 4 The Promise of Liquid Biopsy in Pediatric High Grade Brain Tumours

Liquid biopsy has evolved from a general concept, and later a method of capturing circulating tumour cells (CTCs) in blood samples (Smit and Pantel 2024), to a transformative diagnostic strategy in cancer (Ignatiadis et al. 2021). This approach aims to remotely reconstruct salient and clinically actionable elements of the disease biology from tumour‐derived analytes present in body biofluids, such as blood, CSF, saliva, urine, tears, or secretions (Ignatiadis et al. 2021; Siravegna et al. 2017). In this manner, liquid biopsy circumvents the invasiveness and sampling errors associated with tissue biopsy, at the same time allowing longitudinal assessment of the disease dynamics in real time. All cancer cells with access to a common fluid space would be expected to contribute to the pool of liquid biopsy analytes, thereby collecting information from multiple disease locations. The widely recognized potential insights enabled by liquid biopsy may include differentiation between cancer progression and pseudo‐progression, molecular make‐up of the disease, markers of tumour subtype, spatial and temporal tumour heterogeneity, mutational status, methylation and evolution of the disease. Liquid biopsy may also enable detection of minimal residual disease and impending relapse, assessment of therapeutic targets and responses to treatment, early signs of the emerging therapeutic resistance, overall prognosis and even early detection of cancer, as well as other aspects of considerable clinical significance (Ignatiadis et al. 2021).

The increasing understanding of biological mechanisms underlying the release of tumour related material into biofluids and the growing accessibility of suitable technologies led to explorations of several different liquid biopsy analytes. They include the aforementioned CTCs (Smit and Pantel 2024), circulating cell‐free DNA (cfDNA), circulating tumour DNA (ctDNA) (Siravegna et al. 2017), extracellular RNA (exRNA) (Murillo et al. 2019), soluble biomarker proteins and metabolites (Gold and Freedman 1965) (Batool et al. 2023), tumour‐educated platelets (TEPs) (In ’t Veld and Wurdinger 2019), tumour‐educated leukocytes (TELs) (Chennakrishnaiah et al. 2018), circulating tumour‐associated endothelial cells (CECs) (Bertolini et al. 2006), and different subsets of EVPs (Al‐Nedawi et al. 2008; Skog et al. 2008). Each of these approaches is associated with unique advantages, as well as challenges, and they may be combined to offer complementary diagnostic information. For example, while detection of oncogenic mutations or methylation patterns in ctDNA is accessible through several sensitive and readily applicable technologies, such as droplet digital PCR (ddPCR), sequencing, or methylation profiling, this material often comes from dying cancer cells and lacks information about the cellular proteome. Such sampling may also not reveal the phenotype of the living cell counterparts from which a portion of ctDNA may be released and provides no direct information as to the TME (Ignatiadis et al. 2021; Siravegna et al. 2017; Nassiri et al. 2020). In contrast, CTCs may possess a comprehensive repertoire of biological features associated with underlying cancer, including genome, transcriptome and proteome, but their scarcity in blood poses technical limitations (Ignatiadis et al. 2021) and it may underrepresent the true diversity of cancer cell populations (Rak and Strzadala 2023). On the other hand, at least some subsets of EVPs, such as small extracellular vesicles EVs are numerous (10^10^/mL of blood) (Johnsen et al. 2019) and rich in molecular information reflective of their originating cells (protein, lipids, metabolites, RNA, DNA), but they are also highly heterogenous and challenging to analyse in a simple and reproducible manner (Yu et al. 2021). Despite these obstacles, research in the space of liquid biopsy in cancer remains extremely active with over 12,000 published papers to date and several CTC‐ and ctDNA‐based platforms approved for clinical use (Ignatiadis et al. 2021), along with the emerging battery of EV‐based tests, some of which were recently granted Breakthrough Device designation by the Food and Drug Administration (FDA) (Margolis et al. 2022) (Yu et al. 2021).

Liquid biopsy is also a subject of intense exploration in brain tumours, including CNS malignancies in children (Ricklefs et al. 2024; Berzero et al. 2023; Bonosi et al. 2022; Ray and Vohra 2022; Soffietti et al. 2022; Eibl and Schneemann 2021). The ongoing efforts encompass several pediatric brain cancers (Tripathy et al. 2022; Bonner et al. 2018; Bounajem et al. 2020; Tang et al. 2020) with studies investigating liquid biopsy approaches in medulloblastoma (Buccilli et al. 2024; Albert et al. 2021], ependymoma (de Bont et al. 2008), atypical teratoid/rhabdoid tumour (Kao et al. 2005), in comparative settings of different histopathologies (Pagès et al. 2022), as well as in the space of pHGG (Dietz et al. 2020). The latter diagnosis has been a subject of studies seeking liquid biopsy detection of *H3K27M* mutations (Cantor et al. 2022) along with multiple other traits (*H3.3G34R/V, BRAFV600E/D*, as well as mutant forms of *ACVR1, IDH1, TP53, PIK3CA*, and *MYCN*) (Greuter et al. 2022) in ctDNA extracted from either CSF or blood, and subjected to ddPCR or sequencing assays (Tripathy et al. 2022; Miller et al. 2022; Bruzek et al. 2020; Pan et al. 2019; Huang et al. 2017; Martínez‐Ricarte et al. 2018; Stallard et al. 2018; Panditharatna et al. 2018; Izquierdo et al. 2021; Li et al. 2021; Mueller et al. 2019) (Ronsley et al. 2024; Philippe et al. 2019). For example, in a recent study CSF or plasma of young glioma patients with *H3.3K27M* or *BRAFV600E* mutant brain tumours, ctDNA was interrogated for the presence of these respective alterations using ddPCR assays (Madlener et al. 2025). The sensitivity score for H3.3K27M mutation was 84.61% for CSF and reached 73.68% for plasma samples. Interestingly, the mutation detection rate in CSF depended on the site of sample collection, with 100% detection for intraoperative CSF aspirates, 93% for Ommaya reservoir collection, and 66% for lumbar puncture, likely reflecting the significance of the proximity of the fluid space to the tumour (Madlener et al. 2025).

Notable in this context is the recent study by Erez et al. (Erez et al. 2025) who used a novel strategy to detect H3K27M mutant histone proteins in individual circulating nucleosomes of patients with DMG. This approach, referred to as single‐molecule profiling of the epigenetics of plasma‐isolated nucleosomes (EPINUC), is predicated on the idea that in cancer cells there is a molecular excess of mutant histone proteins in comparison to the corresponding DNA. This would make mutant histone proteins potentially more detectable in assays with single‐molecule sensitivity. To this end, authors have used an ultrasensitive chip‐based platform to immobilize and probe individual nucleosomes, which were then interrogated for K27M substitution in histone H3. Expanding this approach the authors also included detection of mutant TP53 at the single molecule level, and correlated their findings with the subtype of pHGG in a patient cohort containing 52 cases of DMG (Erez et al. 2025).

In some cases, blood or CSF of pediatric brain tumour patients have also been tested for tumour‐related soluble proteins, such as VEGF (Sobol‐Milejska et al. 2017), Cyclophillin A (CypA) or dimethylarginase 1 (DDAH1) (Saratsis et al. 2012), as described and extensively reviewed elsewhere (Tripathy et al. 2022; Dietz et al. 2020; Greuter et al. 2022; Azad et al. 2020; Lu et al. 2019) (Patel et al. 2024). Also, while pathological changes in pHGG are usually confined to the cranium, some explorations of vimentin‐positive CTCs in the blood of patients with *H3.3K27M* mutant brain tumours have also been published recently (Zaky et al. 2023). In settings of pHGG, EVPs have thus far attracted relatively less attention than other liquid biopsy analytes. This may change, however, as the emerging biology of these particles offers a compelling rationale to explore their biological roles and potential diagnostic utility (Singhto et al. 2024).

## 5 Implications of the Emerging Biology of EVPs in Cancer

EVP is a collective designation of all particulate components of the cellular secretome released under health, disease, and death conditions. While EVPs were previously regarded merely as functionless cellular ‘debris’ (Lee et al. 2011), developments of the past several decades revealed their hitherto unsuspected biology along with intricate and diagnostically compelling landscapes. As mentioned earlier the term EVPs comprises two different classes of particles, one consisting of vesicular fragments of cellular membranes, and therefore referred to as extracellular vesicles (EVs). The other subset contains small, membrane‐less, non‐vesicular, extracellular clusters of macromolecules and is thereby defined as extracellular particles (EPs, or non‐vesicular extracellular particles—NVEPs) (Buzas 2023; Couch et al. 2021).

**FIGURE 1:**
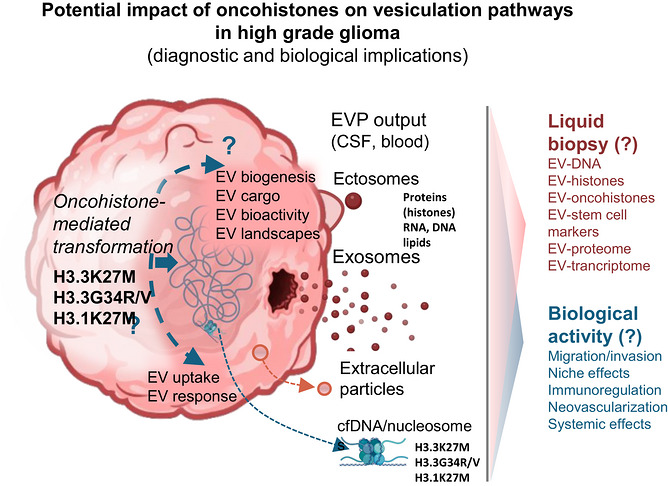
**Potential impact of oncohistones on vesiculation pathways in high grade glioma**. The postulated link between oncohistone‐mediated cellular transformation in high‐grade pediatric glioma may include various aspects of the repertoire of extracellular vesicles and particles. Indicated histone H3 mutations in DMG or DHG cells likely influence EVP biogenesis, subtypes (exosomes, ectosomes, extracellular particles) cargo, and biological properties. These effects may be detectable in biofluids for the purpose of liquid biopsy applications and may also carry functional consequences for the disease, as EVPs interact with other cells and transfer their molecular content—see text for details (the image was created with the use of BioRender).

EVs are highly heterogenous in terms of their physical structures, molecular composition, biogenetic origin and function. Indeed, a large proportion of EVs originates within the cellular multivesicular endosome/body (MVB) through the formation of small intraluminal vesicles (ILVs), which are later released from cells as exosomes (Figure 1). However, EVs may also form at the plasma membrane as structures referred to as ectosomes (van Niel et al. 2018). While exosomes are typically of submicron dimensions (50–150 nm), ectosomes vary dramatically in size from approximately 100 nm for arrestin domain‐containing protein 1 (ARRDC1)‐mediated microvesicles (ARMMs), to 150–1000 nm (microvesicles), to 1–20 µm in diameter (migrasomes, exophers, large oncosomes, or blebbisomes), as extensively reviewed in the recent literature (Buzas 2023; Jeppesen et al. 2023). In addition to EVs released from viable cells, processes of cell death result in encapsulation of cellular remnants in distinct subsets of EVs, including apoptotic vesicles (AVs) and bodies (ABs), which may contain genomic DNA and organelles (Shi et al. 2025). Among EPs, two different subsets described thus far include exomeres (Zhang et al. 2018) and supermers (Zhang et al. 2021), both with sizes below 50 nm and structures comprising distinct assemblies of macromolecules (proteins and nucleic acids) (Jeppesen et al. 2023). While exomeres and supermeres are also released from glioma cells (Xu et al. 2025), at the time of this writing, their biogenesis and biological role in this context have not been described.

Among EVP subsets, exosomes (or exosome‐like small EVs) are the most abundant and most extensively characterized (Buzas 2023). Mechanistically, exosome biogenesis is driven by several tightly regulated processes (Dixson et al. 2023), the most studied of which include the formation of MVBs under the control of endosomal sorting complex required from transport (ESCRT), or through ESCRT‐independent pathways often dependent on syntenin 1 (SDCBP), neutral sphingomyelinase (SMPD3), and through other mechanisms (van Niel et al. 2018). It is noteworthy that some of the molecules mechanistically linked to EV biogenesis, such as syndecan 1 (interaction partner of SDCBP) may distinguish EVs from a broad range of high grade brain tumours (ependymoma, medulloblastoma, diffuse midline glioma, and atypical teratoid/rhabdoid tumour) from EVs isolated from patients with low grade disease, such as pilocytic astrocytoma (Hemmingsen et al. 2026), possibly reflecting a link between EV generating machinery and cellular transformation pathways (Choi et al. 2017).

It should be mentioned that MVB formation may be a prelude to lysosomal degradation of their content, and cellular decisions as to the retention, destruction or extracellular release of ILVs contained within MVBs is tightly controlled. The mechanisms involved in this regulation include intracellular trafficking machineries dependent on the cytoskeleton, small GTPases (Rab7, Rab11, Rab35, Rab27a/b), soluble N‐ethylmaleimide‐sensitive factor attachment protein receptor (SNARE) complexes and other molecules (van Niel et al. 2018; Ostrowski et al. 2010; Martinez‐Arroyo et al. 2021).

The biogenesis of different ectosome subpopulations also includes increasingly well‐defined mechanisms, such as changes in membrane phospholipid asymmetry, as well as blebbing and scission of the plasma membrane during ameboid cell migration. Formation of EVs also occurs within cellular migratory tracks, or through separation of cellular fragments following rapid membrane retraction along with other events, each associated with distinct sets of molecular mediators and diverse ectosome outputs (van Niel et al. 2018; Di Vizio et al. 2009; Yu and Yu 2021; Jeppesen et al. 2025). It should be noted that even after release some of the small EVs may be retained by adhesion molecules at the surface of their originating cell and become released by the regulated action of secretases, such as ADAM10/17, which thereby have a role in control EV landscapes (Bizingre et al. 2025).

Different EV subsets may also exhibit different, and partially redundant, molecular markers. For example, small EVs, are generally enriched in such markers as ESCRT proteins (TSG101), tetraspanins (CD63, CD81, CD9), especially in the case of exosomes and some ectosomes. They are also largely devoid of organelles (van Niel et al. 2018). Annexin A1 is thought be enriched in the case of microvesicles (Jeppesen et al. 2019). Plasma of healthy human blood donors contains EVs from various cells, collectively enriched in ADAM10 and phosphatidyl serine PS (36:1) (Rai et al. 2025), while different cancers may profoundly alter the EV composition in blood (Hoshino et al. 2020). The biogenesis of EPs is still poorly understood, and their emerging markers include metabolic enzymes, such as pyruvate kinase muscle (PKM) and enolase 1 (ENO1) (Zhang et al. 2018) for exomeres, while TGFBI and HSPA13 are enriched in supermeres (Zhang et al. 2021).

Notably, the heterogeneity of EVPs is thought to be far greater than that predicted by the current particle classification. This is because bulk proteomes of purified particle preparations often reveal the presence of hundreds or thousands of proteins, which far exceeds the cargo that individual small EVs or EPs could sterically accommodate. This estimate predicts the existence of multiple *sub*‐subpopulations within EV and EP subsets, a notion that may have important implications in cancer biology and diagnostics (Rak and Strzadala 2023).

Functionally, EVPs are regarded as key components of several cellular processes including: (i) regulated removal (‘dumping’) of superfluous cellular proteins (Pan and Johnstone 1983), DNA (Takahashi et al. 2017) or organelles (Yu and Yu 2021) from viable cells; (ii) encapsulation of remnants of dying cells to prevent them from activating inflammatory responses (Shi et al. 2025); (iii) modification of extracellular microenvironment by deployment of EV‐associated proteolytic activities (Hendrix et al. 2010) or extracellular matrix molecules (Sung et al. 2020); (iv) formation of extracellular hubs of molecular interactions, which could either be a part of homeostatic processes, such as blood clotting, or may result in pathology (Wang et al. 2012; Tolmachova et al. 2007); (v) unconventional secretion of mediators lacking signal sequences, as well as their protection from degradation and delivery across tissue barriers (Morad et al. 2019); (vi) non‐gradient deployment of potent molecular signals to trigger strong focal responses at a distance (Taraboletti et al. 2006); (vii) formation of EV tracks regulating collective directional cell migration (Sung et al. 2020); and (viii) acting as mediators of intercellular communication and molecular exchange over short and long distances. The latter activity encompasses such already documented effects as EV‐mediated tumour‐vascular interactions (Adnani et al. 2022); systemic immunoregulation (Chen et al. 2018; Wortzel 2023), metabolic responses (Wang et al. 2023; Cao et al. 2022), conditioning of tissues for (or against), distant metastasis (Peinado et al. 2012; Wortzel et al. 2019; Plebanek et al. 2017), and multiple other effects.

As mediators of cell‐cell communication, EVs may either interact with surface structures (receptors) of their target cells triggering intercellular signals, or they may become internalized by recipient cells through several mechanisms, such as membrane fusion, endocytosis and macropinocytosis (Mulcahy et al. 2014). The EV internalization is the key mechanism enabling molecular transfer between donor and recipient cells (Al‐Nedawi et al. 2008; Spinelli et al. 2024; Mulcahy et al. 2014; Chennakrishnaiah et al. 2020; Choi et al. 2021). Such EV uptake into cellular interior, may lead to their cargo being delivered to the endosome and subsequently undergo lysosomal degradation, repackaging EVs for secondary release (Luga et al. 2012), endosomal escape into the cytoplasm (O'Brien et al. 2022), transfer into the nucleus (Lorico et al. 2025), or re‐expression of EV membrane proteins on the surface of recipient cells (Spinelli et al. 2024).

EVs are rich reservoirs of informative and functionally important nucleic acids (O'Brien et al. 2022). The EV‐associated RNA profiles are not identical to those of their parental cells due to selective packaging mechanisms (O'Brien et al. 2022), but they do contain cancer‐related repertoires of non‐coding and mRNA sequences (Murillo et al. 2019; O'Brien et al. 2022; Wei et al. 2017), including informative microRNA and transcripts harbouring cancer‐specific mutations (Skog et al. 2008; O'Brien et al. 2022; Noerholm et al. 2012). This material is protected from degradation by EV membranes and thereby diagnostically accessible in relevant biofluids. While the presence of genomic DNA in cancer EVs (Lee et al. 2014) is debated (Jeppesen et al. 2019), recent studies identified EV‐associated chromatin, histones, and DNA, often located on the outer surface (corona) of EVs, or, according to some reports, within the EV lumen (Wortzel et al. 2024; Singh et al. 2025). Cellular stress (Singh et al. 2025) or cancer‐specific molecular mechanisms dependent on APAF1 and NCF1 (Wortzel et al. 2024) were proposed to govern this association. These processes may allow, in some cases, to use EV preparation protocols for cancer‐related DNA enrichment in the context of liquid biopsy applications.

The involvement of EVPs in cancer progression is driven by several upstream mechanisms, including oncogenic transformation (Choi et al. 2017), therapeutic stress (Keklikoglou et al. 2019; Garnier et al. 2018; Montermini et al. 2015), impact of TME (Xu et al. 2018; Han et al. 2019) and cancer‐associated macro‐environment (Wortzel et al. 2019; Al‐Zhoughbi et al. 2014). These linkages are epitomized by the influence of oncogenic mutations on the extent and nature of cancer cell vesiculation (Al‐Nedawi et al. 2008) and on the cargo of tumour‐derived EVs, including their protein (Choi et al. 2018), RNA (Skog et al. 2008) and DNA content (Lee et al. 2014). In a recent study tumour prone mice harbouring TP53 null mutation were shown to exhibit extended survival, if the TP53‐regulated TCTP protein, which is implicated in EV biogenesis, was pharmacologically or genetically inhibited (Amson et al. 2024). Moreover, oncogenic pathways driven by mutant *EGFR, RAS*, and other alterations were shown to impact the magnitude of the EV uptake by cancer cells (Lee et al. 2016; Nakase et al. 2015) and the nature of the uptake mechanisms (e.g. macropinocytosis versus endocytosis) (Choi et al. 2021). Some of these pathways also influence the nature of cancer cell responses to EVs received from the TME, as exemplified by the effects of angiocrine EVs released from the endothelial cell compartment on glioma stem cells (Adnani et al. 2022).

It is also noteworthy that EVs represent a unique portal through which proteins, RNA and DNA harbouring cancer‐specific and overtly oncogenic mutations may be released from tumour cells and enter extracellular fluid space, circulating biofluids and blood. The resulting contact between multiple potential target cells and the transforming or regulatory content of cancer EVs may lead to a range of biological responses, including pro‐inflammatory effects (Wortzel et al. 2024), metabolic changes (Wang et al. 2023) or horizontal quasi‐transformation‐like states associated with the transfer of oncogenic cargo (Al‐Nedawi et al. 2008; Spinelli et al. 2024; Lee et al. 2016). It has been reported that ‘grafting’ cancer‐related mutant oncogenic molecules into intracellular signalling networks of EV recipient cells may alter their phenotype, function, and viability (Spinelli et al. 2024; Peinado et al. 2012; Chennakrishnaiah et al. 2020; Lee et al. 2014; Tominaga et al. 2015). At the same time, the existence of EV‐associated extracellular pools of macromolecules harbouring cancer‐specific traits, or co‐expressed in a unique fashion along with markers of cellular identity and function, represent an attractive opportunity to develop EV‐based liquid biopsy applications (Rak 2013; Al‐Nedawi et al. 2008; Skog et al. 2008; Yu et al. 2021) including in pHGGs.

## 6 EVPs as Emerging Liquid Biopsy Analytes in Brain Cancer

Over the past nearly two decades, considerable research efforts were devoted to the development of EV‐based liquid biopsy biomarkers for adult HGG (Al‐Nedawi et al. 2008; Skog et al. 2008; Ricklefs et al. 2024; Bonosi et al. 2022; Soffietti et al. 2022; Salviano‐Silva et al. 2025; Batool et al. 2024; Balana et al. 2022; Bettegowda et al. 2025). While such platforms are still to reach clinical utility, their experimental explorations offered valuable lessons as to the potential of EVs as liquid biopsy analytes in the space of brain tumours (Berzero et al. 2023; Santiago‐Dieppa et al. 2014; Marei et al. 2022). Thus, while EVs are abundant in biofluids, carry and protect from degradation a rich cancer‐related repertoire of lipids, proteins, RNA, and DNA, and are able to traverse the blood brain barrier (Morad et al. 2019), only a small fraction of EVs found in plasma is actually derived from cancer cells themselves. Indeed, the majority of plasma EVs originates from platelets, blood cells, endothelial cells, and other tissues (Berzero et al. 2023; Rai et al. 2025; Holcar et al. 2025). This ‘dilution’ effect renders detection of certain cancer‐specific EV cargos challenging and prone to fluctuations (Skog et al. 2008; Berzero et al. 2023). It should also be noted that cancer patients experience considerable changes in the total numbers of circulating EVs, such that in GBM the number of EVs in plasma could be at least three‐fold higher than in cancer free individuals (Osti et al. 2019). This increase may, again, include the contribution of EVs from different tumour‐associated, non‐transformed cell populations (tumour stroma, microenvironment, blood cells and organs), including T cells (Whiteside 2023) and the myeloid cell compartments. However, these ‘host’ cell EVs may have an independent diagnostic value, as a reflection of systemic responses to the disease (Tsamchoe et al. 2023). Additional diagnostic clues may also be extracted from the heterogeneity of EVs and NVEP produced by cancer cells themselves (Rak and Strzadala 2023). In this regard recent studies describe the intricate repertoire and molecular cargo of glioma derived EVs and NVEP (exomeres and supermeres) contained in the secretome of GBM cells (Xu et al. 2025).

While plasma EVs harbour clinically relevant information, they often pose considerable analytical challenges in terms of reliable detection of pre‐determined molecular targets. For cancer‐derived EVs, the limit of detection may depend on both the overall abundance of the molecule in question, its linkage to EV biogenesis machinery, and the technology being used for its detection, with PCR‐based or sequencing strategies usually affording a greater sensitivity of EV‐associated nucleic acids (Batool et al. 2024). In this regard, proximal fluids, such as CSF may offer a richer source of tumour‐derived EVs along with a less complex host EV background (Chen et al. 2013; Rudà et al. 2024; Del Bene et al. 2022; Reetz et al. 2025).

Analysis of small, nano‐sized EVs may require specialized protocols, instrumentation, and technologies (Shao et al. 2018) to reliably detect, quantify, and profile their molecular content (Welsh et al. 2024). While the short half‐life of EVs in the circulation (often minutes (Wang et al. 2012)) may be an advantage, offering a more dynamic picture of underlying biological changes, the levels of circulating EV may fluctuate depending on their biogenesis and phagocytic bioelimination, mostly occurring in liver and spleen (Hyenne et al. 2019). Using sensitive molecular tracers enabled demonstration of different time courses of bioelimination of externally administered EV, which were cleared with different kinetics from different biofluids for up to several hours (Lai et al. 2014). In experimental organisms such as zebra fish or mice endogenous small EVs tend to be captured by endothelial cells and macrophages (Verweij et al. 2019). In another study EVs derived from mutant RAS‐driven tumours in mice were taken up by circulating white blood cells, which retained their cargo within the circulation for days (Chennakrishnaiah et al. 2018). Notably, platelets have been proposed to act as reservoirs of cancer derived EVs and their oncogenic cargo (Nilsson et al. 2011). Recent studies have also suggested that EVs may rapidly bind to red blood cells and platelets, and exhibit different biodistribution patterns depending on the properties of EVs, their carrier cells and organismal processes, such as inflammation (Pavlova 2025). Although cancer‐specific molecular cargo of EVs, such as mutant proteins or nucleic acids can offer valuable diagnostic information, they may be present only in a fraction of tumour derived EVs. For example, mesenchymal glioma stem cells highly and uniformly positive for mutant EGFRvIII, release this receptor as cargo of small EVs, which however is packaged into less than 50% of all tumour derived EVs (Pishavar et al. 2026).

Many of these challenges are being addressed by increasingly refined analytical strategies. Thus, the detectability of informative cargo of EVs can be enhanced through the use of ultrasensitive technologies, such as amplifiable fluorescent probes (de Voogt et al. 2021), improved instrumentation (Kim et al. 2024), and new detection protocols (Shao et al. 2015), as exemplified by the recently developed CRISPR‐based SCOPE platform (Song et al. 2024). While EV heterogeneity and complexity may be regarded as baffling problems, the advent of technologies operating at a single vesicle level opens the entirely new range of possibilities for at least two reasons. First, single vesicle platforms may detect very rare, but highly cancer‐specific EV subpopulations. Second, these approaches may reveal complex landscapes of EV subpopulations, and these patterns could be diagnostically informative in their own right (Rak and Strzadala 2023).

**FIGURE 2:**
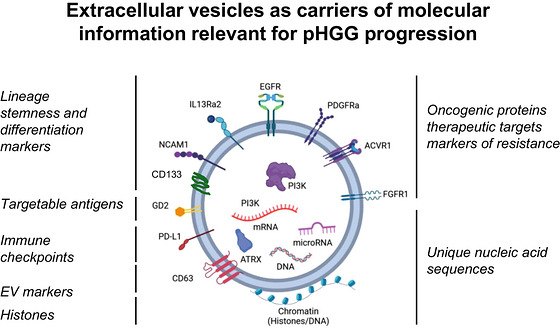
**Extracellular vesicles as carriers of molecular information relevant for pHGG progression**. Molecular elements of HGG biology that are likely incorporated into EV cargo (proteins, nucleic acids)—see text for details (the image was created with the use of BioRender).

For example, technologies interrogating the co‐expression of several EV cargo molecules, each endowed with a limited cancer specificity, may result in cancer‐specific combined, multimolecular phenotypes of individual EVs, or their subpopulations (Wallucks et al. 2024). On the other hand, improved chip‐based and nano‐flow cytometry platforms may allow increasingly precise multiplexing of EV cargo and mapping of their informative subsets (Kim et al. 2024). In addition, label‐free technologies, such as surface enhanced Raman spectroscopy (SERS) offer a molecularly agnostic high dimensional EV mapping capacity with high resolution. One example of such technology is the recently developed MoSERS platform that enables capturing and profiling of individual EVs, followed by reassembly of their landscapes using machine learning algorithms (Jalali et al. 2023). Using this platform on plasma EVs enabled accurate separation of patients with GBM from healthy controls (Jalali et al. 2023). With increasing dimensionality of single EV technologies these approaches may eventually provide tools to exploit rather than circumvent EV heterogeneity. Thus, not unlike in the case of single‐cell sequencing technologies (in microscale), mapping diagnostically informative complex EV landscapes (in nanoscale) may lead to more granular information about the underlying disease processes. In other words, EV patterns in settings of liquid biopsy diagnostics may serve as biological barcodes of complex disease states under investigation (Rak and Strzadala 2023) (Figure 2). Several technologies are being explored to implement such approaches in brain tumour diagnostics and adapting them to pHGG would be of great interest.

## 7 The Road to EV Application in Liquid Biopsy of Pediatric HGG

Studies on the roles and diagnostic applications of EVs in pHGG are presently limited. However, there are experimental indications that, similarly to adult HGG, also in pediatric tumours EVs may be both a part of the disease biology and a prospective analyte for liquid biopsy applications (Table 1). Thus, using OPBG‐GBM‐001 cells harbouring *H3G34R* mutation and derived from a DHG lesion, Petrini et al. have demonstrated the release by cancer cells of EVs detectable by super‐resolution (STED) microscopy (Petrini et al. 2025). In another study, two pHGG patient‐derived cell lines originating from either DHG (OPBG‐GBM002), or DMG‐H3K27‐mutant (OPBGDIPG002) lesions were analysed for inter‐clonal communication involving EVs and impacting cell motility. Notably, interference with EV biogenesis using the neutral sphingomyelinase inhibitor (GW4969) resulted in impeded cell migration, suggesting the role of EVs in this process (Pericoli et al. 2023). This observation is also in line with suggestions as to the role of DMG EVs in tumour cell invasion (Kluiver et al. 2020). EV‐associated small RNAs profiles were recently studied in a series of three DMG‐K27M‐mutant cell lines along with medulloblastoma cells, leading the authors to suggest that EV transcriptome is largely depleted for microRNA and enriched for Y‐RNA (Magaña et al. 2022). Nonetheless, mRNA target analysis based on detectable spectrum of EV‐associated microRNA revealed enrichment for several cancer‐related pathways (Magaña et al. 2022). In another study, Shan et al. used functionalized macrophage EVs to deliver histone deacetylase (HDAC) inhibitor, Panobinostat, or siRNA against TP53‐induced protein phosphatase (PPM1D) into DMG cells xenografted into mice (Shan et al. 2022). These authors reported that EV‐based nanodrugs improved the survival of tumour‐bearing mice. Indeed, EV‐mediated interactions in DMG have become an area of increasing therapeutic interest (Chen et al. 2023).

**TABLE 1:** Examples of Studies on the role of Extracellular vesicles in pediatric high‐grade glioma.

Study (ref)	pHGG	EV/EP source	Molecular mediator or cargo	Observations
Pertini et al. (Petrini et al. 2025)	DMG‐H3G34 mutant cells	Cell culture	Not studied	pHGG cells release EVs detectable by superresolution microscopy
Pericoli et al. (Pericoli et al. 2023)	DHG and DMG cell lines	Cell culture	EVs effects were inhibited by GW4869	Blockade of EV biogenesis impacted cancer cell motility
Magana et al (Magaña et al. 2022)	DMG‐H3K27M mutant cells	Cell culture	EV associated RNA biotypes	EV‐associated microRNA target cancer pathways
Oza et al. (Oza et al. 2025)	DMG‐H2K27M mutant cells	Cell culture (intact or irradiated)	Proteome Transcriptome	Molecular transfer of radiation resistance between DMG cell subpopulations
Shan et al (Shan et al. 2022)	DMG	Macrophages	Panobinostat PPM1D‐siRNA	Macrophage EVs carrying Panobinostat or PPM1D‐siRNA improved survival of mice harbouring DMG xenografts
Fernandez Garcia et al. (Iyer et al. 2026)	pHGG cell lines	Cell culture	Radial glia EV‐associated protein signature (PTPRZ1/ GLAST)	Phenotyping of EVs from radial glia‐like cells that correlated with pHGG features
Chen et al. (Chen et al. 2023)	DMG	Therapeutic EVs	Therapeutic cargo	Design of functionalized EVs to treat brain stem tumours
Tuzesi et al. (Tűzesi et al. 2017)	pHGG stem cell lines	Cell culture	microRNA; miR‐ 1290 and miR‐1246 linked to‘stemness’	Pediatric HGG cells release EVs containing abnormal compositions of microRNA
Buzova et (Buzova et al. 2025)	DMG‐H3K27M patients and cell lines	Histone complexes (particles?)—EVs not directly implicated	Histone complexes in plasma, CSF and cell culture media	In DMG patients altered composition of histones and their complexes is detectable in biofluids

Building on the links between oncogenic signalling, EV cargo, and EV mediated cellular reprogramming, Oza et al. (Oza et al. 2025) explored the impact of EVs on the biology and radiation resistance of *H3K27M*‐mutant pDMG cells. In this setting the uptake of small EVs derived from intrinsically radioresistant H3K27M‐pDMG cells by their radiosensitive DMG counterparts resulted in a horizontal transfer of radioprotective properties. These responses were associated with reprogrammed metabolism of recipient cells by increasing their glycolytic and tricarboxylic acid cycle intermediates, coupled with upregulation of genes involved in oxidative phosphorylation, along with enhanced DNA damage repair capacity, as evidenced by elevated 53BP1 expression and reduced γH2AX foci following irradiation (Oza et al. 2025). This study highlights a possible role of small EVs in therapy resistance in pHGG, in line with analogous and previously reported findings in adult GBM and other cancers (Yekula et al. 2021). While these are limited and incipient experimental inroads, they collectively reinforce the notion that, similarly to other brain tumours, pHGG cells produce EVs with relevant molecular cargo and notable functional properties, and thereby they may be ripe for diagnostic explorations.

Despite this increasingly solid rationale, at the time of this writing there were no studies directly demonstrating the applicability EVs as liquid biopsy analytes in pHGG patients. However, early insights began to emarge. For example, an interesting exploration of this possibility was recently offered by Fernandez Garcia who studied EVs derived from radial glia (RG)‐like cancer cells, which molecularly resemble pHGG. These investigators aimed to develop a molecular signature of pHGG cells and identified co‐expression of proteins (PTPRZ1/GLAST) that was subsequently validated, and further studies revealed the existence of EV‐associated relevant therapeutic targets (GD2) (Fernandez Garcia  et al. 2026).

Nonetheless, the majority of studies thus far cited in this domain rely on findings obtained in adult HGG, to suggest the feasibility of EV‐based biomarker discovery in pediatric glioma (Singhto et al. 2024; Chen et al. 2013; Yin et al. 2019; Yue et al. 2019; Zeng et al. 2018), but direct evidence to this effect is presently lacking. One way to fill this gap before robust experimental studies become available is to consider directions set by EV‐based liquid biopsy research conducted up to this point in other cancers, and relate them to the unique biology of pHGG. Thus, one of the major tenets linking cancer progression to diagnostically informative EV profiles is the aforementioned notion that unique fingerprints of oncogenic drivers, such as mutant proteins (Al‐Nedawi et al. 2008), RNA (Skog et al. 2008) and DNA (Lee et al. 2014; Balaj et al. 2011; Kahlert et al. 2014; Thakur et al. 2014) are often found in cancer‐derived EVs. This raises the question as to a possible inclusion of oncohistones in the cargo of EVs derived from DMG, or DHG tumours, in a similar manner as histones become incorporated into EVs of other cancers (Wortzel et al. 2024; Singh et al. 2025). Evidence to this effect is being actively sought (Jahouri et al. 2025).

Since oncogenes such as EGFR (Al‐Nedawi et al. 2008; Choi et al. 2018), RAS (Choi et al. 2021; Lee et al. 2014), MYC, AURKA (Kilinc et al. 2021) and others alter EV biogenesis and EV cargo loading pathways, thereby affecting both the EV release profiles and their molecular content (Choi et al. 2017), it is plausible that landscapes of EV released from pHGG cells may also carry the unique signature of these tumours, or of oncohistone mutations themselves (Figure 1), in terms of protein composition, RNA content (including microRNA and small RNA biotypes), and the presence of DNA (beyond the inclusion of oncohistone mutations themselves).

Indeed, the linkage between oncogenic transformation and non‐cell autonomous microenvironmental responses mediated by altered secretomes of mutant cancer cells has emerged several decades ago (Rak et al. 1995) and became a useful paradigm in interpreting the abundance and recruitment of vascular, myeloid, immune and stromal cells to the sites of tumour growth (Yuan et al. 2024). For example, the *H3.3G34W* mutation found in giant cell tumours of the bone (GCTB) (genetic lesion reminiscent of oncohistones associated with DHG) leads to changes in the seceretory phenotype of cancer cells and robust stromal cell responses (Khazaei et al. 2020). In GBM up to 30%–40% of the tumour mass consist of non‐cancer cells (Quail and Joyce 2017) and this is particularly accentuated in the mesenchymal‐subtype of these tumours enriched for *NF1* mutations (Neftel et al. 2019). In EGFR‐driven xenografts of GBM, EVs harbouring onocogenic EGFR were found to switch angiogenic vascular growth pattern to an alternative neovascularization process (vasectasia) (Spinelli et al. 2024). It is tempting to speculate that similar effects may also occur downstream of oncohistones in pHGG, and their understanding may offer both biological and diagnostic insights. This, again, is a subject of active investigation (Jahouri et al. 2025; Tawil et al. 2025).

**FIGURE 3:**
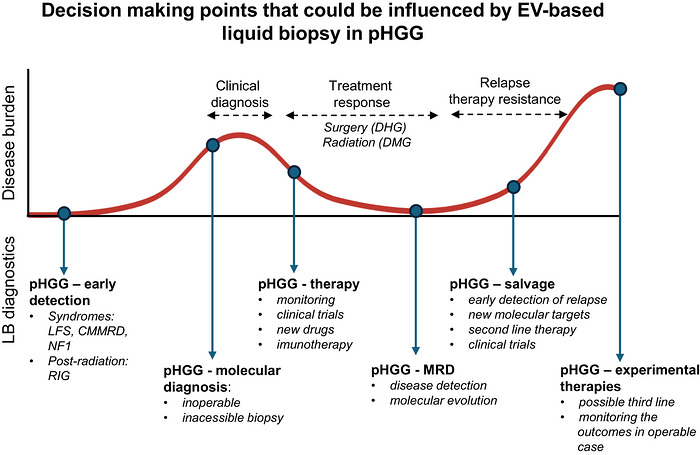
**Decision making points that could be influenced by EV‐based liquid biopsy in pHGG**. Several stages in the natural history of pHGG could be monitored using liquid biopsy, including the analysis of EVs in blood or cerebrospinal fluid. These approaches could reveal molecular evolution of the disease, emerging therapeutic targets, responses to experimental therapy and other aspects (the image was created with the use of BioRender).

The possible role of EVs as companion diagnostics is also worth considering in the context of pHGG. Relevant to this context is the observation that cellular stresses, such as those induced by radiation, perturb EV biogenesis, leading to increased production of small, exosome‐like EVs (Yu et al. 2006). Targeted agents (Montermini et al. 2015) and chemotherapy (Keklikoglou et al. 2019) also profoundly impact EV landscapes. Therefore, it would be of great interest to assess whether responses to radiation, targeted therapies, such as those currently under development (Doraviprone, Panobinostat and others) (Arrillaga‐Romany et al. 2024; Monje et al. 2023), or experimental immunotherapy (Monje et al. 2025) would be reflected in profiles of EVs accessible in biofluids of pHGG patients. In this regard, EVs reflecting stromal and systemic responses to pHGG progression could also be integrated within liquid biopsy assays. Naturally, single EV profiling technologies, such as those based on fluorescent probes (Wallucks et al. 2024; Lee et al. 2018) or label‐free platforms, such as MoSERS (Jalali et al. 2023), would enable harnessing EV heterogeneity, as a reflection of treatment response (Figure 3). In parallel, mathematical approaches and statistics are essential to translate these high‐dimensional EV measurements into quantitative, interpretable, and robust heterogeneity signatures. With progress in nanotechnology and applied deep learning, many of these questions are likely to be addressed in the relatively near future.

## 8 Concluding Remarks

The management of pHGG is increasingly predicated on molecular diagnosis, as is the development of new targeted agents and immunotherapies, efforts which may hopefully break the current therapeutic gridlock. Meeting these objectives mandates creating an access to molecular repertoires of these aggressive tumours, to follow their evolution in real time. While in expert centres tissue biopsy is a norm in brain tumour diagnosis, and it is also increasingly feasible in DMG, these advances face some important limitations that could be alleviated by liquid biopsy (Ignatiadis et al. 2021; Tripathy et al. 2022), with EVPs as prospective analytes.

Due to their poor surgical accessibility (especially DMGs), vulnerability of patients and a dire need to develop effective systemic therapies and companion diagnostics liquid biopsy research in pHGG is especially unique. However, the promise of exploiting the role of EVPs in the biology of pHGGs, and as corresponding liquid biopsy analytes to monitor different aspect of these devastating diseases is still curtailed by significant gaps in knowledge. As this brief overview indicated, the body of solid data as to the ‘vesiculome’ associated with this class of tumours is relatively limited and the purpose of this discussion is to build on these preliminary data and the emerging research directions to better articulate and refine the questions that could guide the immediate and long term investigative efforts. Among important unknowns in this regard is the uncertainty as to the exact spectrum of particulate entities (subsets of EVs and NVEPs) that pHGG cells and their non‐transformed cellular neighbours export into the TME, proximal biofluids and circulation. What are the mechanisms of this secretion and what biological messages does it contain? How could diagnostic information be best extracted from this likely rather complex repertoire, and which particles carry the key analytes (e.g. oncohistones)? Does the diversity of EVs and EPs collectively contains the most complete reflection, fingerprints or barcodes of the disease? (Broekman et al., 2018)

Addressing these questions would require considerable investments in overcoming methodological challenges, some unique to HGGs (models, accessible research material) some more general in nature. The latter includes still insufficient conceptual and technical grasp of EVP heterogeneity, which could be expanded through the use of single particle technologies (Shao et al. 2018; Pishavar et al. 2026; Carney et al. 2024), combination of complementary analytical methods (Robinson et al. 2026) and machine learning (Jalali et al. 2023). Clinically meaningful deployment of these approaches would also require close attention to pre‐analytical variables (Bettegowda et al. 2025), assay robustness to minimize technical variability, as well as measures to increase standardization and reproducibility across different centres, an ongoing effort supported by building consensus guidelines and promoting best practices in the field (Welsh et al. 2024; Hendrix et al. 2023; Lucien et al. 2023; Welsh et al. 2020).

Until a more extensive preclinical and clinical testing is carried out, the EVP‐based liquid biopsy in pHGG may face similar uncertainties as those encountered in other cancers. While the biology of EVPs and especially their unique collective ability to reflect molecular make up of their cellular sources, as well as large numbers found in biofluids offer unprecedented opportunities, it is likely the practicalities of specific assays and their value in clinical decision making that would ultimately define the place of EVPs in the field in liquid biopsy diagnostics. While this is very much an ongoing process, the rapid development of biological foundations, nanotechnologies and machine learning approaches raise hopes as to the future role of EVP analysis in cancer care (Jalali et al. 2023), including in pHGGs.

As in all brain tumours, the role of blood brain barrier (BBB) and brain tumour barrier (BTB) (Jain et al. 2007) raise questions as to the extent to which EVPs generated by cancer cells and stroma may enter systemic circulation and become analytically accessible. In this regard three major research directions are already underway: investment in assay sensitivity, accessing proximal biofluids for EVP sampling (e.g. CSF) and promoting technologies that may facilitate the exit of EVPs from the tumour milieu into the circulation. The latter includes focused ultrasound (FUS) that may temporarily disrupt vascular barriers enabling the entry of liquid biopsy analytes, including EVPs, from the tumour mass into the circulation (Meng et al. 2021; Kline‐Schoder et al. 2025). Other techniques to control BBB are also being studied (Adnani et al. 2025).

The advent of liquid biopsy across cancer indications, including some early inroads in pediatric solid tumours (Janssen et al. 2024) and in pHGG (Dietz et al. 2020), is largely predicated on technological improvements, choice of intuitively appealing analytes, such as ctDNA (presently), and their empirical explorations. However, increasingly, the biology of liquid biopsy analytes becomes a valid consideration. For example, circulating ctDNA may either reflect remnants of dead cancer cells that no longer exist, or a cargo of EVs from viable cancer cell populations, with specific properties. DNA may be loaded onto cancer EVs as a result of the oncogenic transformation (Lee et al. 2014), stress (Singh et al. 2025) or expression of specific genes, such as APAF1 or NPC1 (Wortzel et al. 2024). Each of these mechanisms may have a different diagnostic meaning in the context of liquid biopsy. Specific subsets of EVs may also reflect different biological aspects of the disease, such as migratory behaviour of tumour cells (Albert et al. 2021; Di Vizio et al. 2009) or organismal responses to cancer progression (Tsamchoe et al. 2023). Understanding what each of these aspects signifies clinically is worth exploring in parallel to translation of known approaches to the clinic. Indeed, lessons from the unique biology of pHGG subtypes may offer important clues as to new directions of liquid biopsy in this setting, including the role of EVPs.

## Author Contributions

**Livia Garzia**: conceptualization, writing – review and editing, supervision, funding acquisition. **Nada Jabado**: supervision, conceptualization, writing – review and editing, funding acquisition. **Samia Jahouri**: conceptualization, writing – original draft, writing – review and editing. **Mahsa Jalali**: conceptualization, writing – review and editing. **Laura Montermini**: conceptualization, writing – review and editing. J**anusz Rak**: conceptualization, writing – original draft, writing – review and editing, funding acquisition, supervision. **Nadim Tawil**: conceptualization, writing – review and editing.

## Funding

This work was supported by the liquid biopsy program sponsored by Fondation Charles Bruneau and Fondation CIBC. J.R. is a recipient of the Jack Cole Chair in Pediatric Hematology/Oncology. Support was also received through operating grants from Canadian Institutes for Health Research, Cancer Research Society, New Frontiers in Research Fund—Exploration and infrastructure funds from Canada Foundation for Innovation and Canada First Research Excellence Fund—D2R

## Ethics Statement

The authors have nothing to report.

## Conflicts of Interest

All authors declare no conflict of interest

## Data Availability

The authors have nothing to report.
